# Dr. Dharmendra's Enduring Influence on Leprosy: From Laboratory to Disease Control

**DOI:** 10.7759/cureus.69459

**Published:** 2024-09-15

**Authors:** Mohammed S Hyder, Arun Inamadar

**Affiliations:** 1 Dermatology, Shri B. M. Patil Medical College Hospital and Research Centre, Bijapur Lingayat District Education (BLDE) (Deemed to be University), Vijayapura, IND

**Keywords:** historical vignette, infectious disease, lepromin, leprosy, national leprosy eradication programme

## Abstract

A stalwart of Indian dermatology, Dr. Dharmendra was particularly known for his breakthrough research on leprosy. Spanning over several decades, his work was marked by significant contributions, including the development of Dharmendra's lepromin, which served as a critical diagnostic tool and in advancing the understanding of immunological aspects of leprosy. Born in 1900 in present-day Pakistan, his early education and work experiences set the foundation for his later achievements in leprosy research. Despite the social stigma associated with the disease at the time, Dr. Dharmendra's dedication led him to take up key roles, such as the first director of the National Leprosy Control Programme and head of the Central Leprosy Teaching and Research Institute in Chengalpattu. His work significantly impacted leprosy control efforts in India. ⁤⁤His legacy was further cemented by his leadership in founding the Indian Association of Leprologists and his tireless editorial work in the Indian Journal of Leprosy. Through his publications and ongoing research, even after retirement, he continued to influence the field. Dr. Dharmendra's life and work remain a cornerstone of leprosy research, inspiring future generations. His legacy continues to be felt in the ongoing efforts to combat leprosy.

## Introduction and background

**Figure 1:**
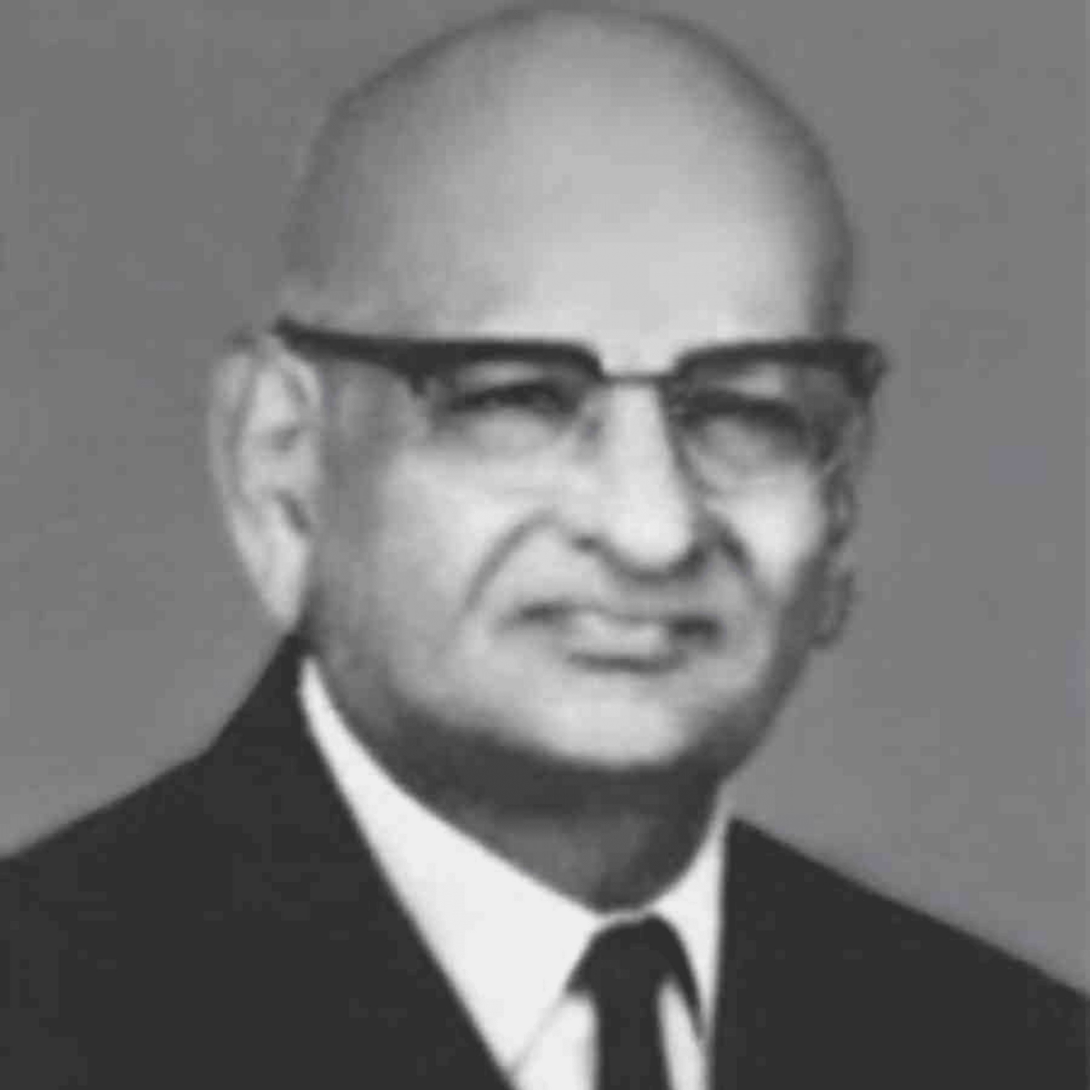
Dr. Dharmendra (1900-1991) Source [1]

Dr. Dharmendra (Figure 1), a trailblazing figure in Indian dermatology, transformed the discipline with his innovative work on leprosy, in particular, the discovery of Dharmendra's lepromin, a critical tool in the understanding of this ancient illness. His efforts expanded medical knowledge while also laying the groundwork for modern leprosy control programs in India, making an everlasting mark on leprosy history. His career spanned several decades and was defined by his unwavering quest for one of humanity's oldest ailments. He was instrumental in developing the strategies as the first to head the National Leprosy Control Programme, eventually resulting in a major drop in the prevalence of leprosy in India. His novel technique of lepromin testing not only revealed new information about the disease's immunological characteristics but also provided a diagnostic breakthrough. Apart from his technical accomplishments, Dr. Dharmendra's leadership at the Central Leprosy Teaching and Research Institute in Chengalpattu established new benchmarks in medical research and public health management. His work continues to influence modern dermatological techniques and is a cornerstone of leprosy research globally.

## Review

Early life and education

Born in present-day Pakistan in 1900, Dr. Dharmendra studied in several schools owing to his father's frequent transfers and ultimately passed the matriculation examination from Punjab University and went on to join the intermediate science course at the Government College in Lahore with the aim of becoming a doctor. Disappointed with being unable to join his desired course at King Edward Medical College, he joined the Bachelor of Science course in botany. In his second year, he applied for admission into the medical college, and on account of his high merit, he was admitted into the medical course [2].

Participation in the non-cooperation movement

In his second year of medical course, Dr. Dharmendra left his studies to join the non-cooperation movement on the call of Mahatma Gandhi after the tragedy of the Jallianwala Bagh massacre. After idling away for a year, there was no longer a requirement for students to leave the colleges for the non-cooperation movement. He then reapplied for medical college, and yet again, he was accepted. Thus, he lost two years of his life before going ahead and, in due course of time, passed the MBBS examination from Punjab University [2].

Early work opportunities

After getting an opportunity to work in a laboratory, he honed his skills in Delhi and Shimla for two years, learning laboratory techniques. However, the routine work soon became monotonous, prompting a desire to join a research institute. This led him to take up a position at the School of Tropical Medicine, Calcutta. His first assignment was to work on the Zondek-Ascheim test for the detection of early pregnancy and to indicate the sex of the fetus in mice; its success boosted his confidence and earned praise from the director [2]. His second assignment involved asthma research, revealing that not all cases of asthma were due to sensitivity but rather due to chronic bronchitis, which required different treatments. He then conducted pneumococcal typing on pneumonia patients [2].

Interest in leprosy work

A renewed sense of national duty led him to focus on leprosy research, recognizing the need for Indian experts in a field dominated by foreigners. Despite family opposition due to the stigma associated with leprosy, he commenced work in the department of leprosy at the School of Tropical Medicine, involving clinical, laboratory, and immunological research. Dr. Dharmendra studied an immunological test conducted in vivo called the lepromin test, first introduced by Dr. Mitsuda of Japan. The active principle in the leprosy bacillus that caused this positive reaction was sought, and with the crude instruments available to him in the department, he succeeded in the discovery of the active principle, which was the lepromin moiety of the leprosy bacillus responsible for the early and late lepromin reactions. This discovery was considered so important that the supervisor suggested a joint publication in both their names, but Dr. Dharmendra declined this. Despite initial fears of displeasure, the supervisor became more amicable and appreciative of his work. Dr. Sengupta at the Japanese Leprosy Mission for Asia (JALMA), Agra, later confirmed all findings regarding the active protein responsible for lepromin positivity. More recently, almost four decades after his discovery, the International Federation of Anti-Leprosy Associations (IMMLEP) project of the World Health Organization (WHO) worked on this project and reached the same conclusion regarding the active constituent of bacillus responsible for lepromin test positivity. However, it is regrettable that none of the IMMLEP workers referenced the work done in 1941, much earlier than the inception of their project. However, Dr. Jopling, in his book, gave due credit to Dr. Dharmendra's work, which was made possible by the availability of a large number of leprosy bacilli from artificially infected armadillos [2].

Development of Dharmendra's lepromin

His profound interest in the lepromin test and seeking the active principle led him to perform another test on a project basis. Due to the unavailability of the protein solution required, he utilized his own method, called the chloroform method, to isolate the bacilli from the tissue. He then isolated the bacilli, dried it in a vacuum, and prepared this vaccine in carbol saline. This vaccine was called Dharmendra's antigen due to personal reasons and is now referred to as Dharmendra's lepromin. Dr. Dharmendra was standardizing this lepromin by the weight of the dry powder of the leprosy bacilli and, on Dr. Sengupta's advice, modified it to count the bacilli rather than to standardize by the weight of the dry powder of leprosy bacilli to get consistent results [2].

Prophylactic dapsone in contacts of leprosy patients

Dr. Dharmendra and his colleagues framed and carried out the scheme of administering prophylactic dapsone to contacts of leprosy patients, whereby they identified a large number of contacts of leprosy patients and conducted this study and found that it provided 52% protection from infection in adult contacts of patients of leprosy and even more in children aged up to 12 years. Later, they introduced a depot preparation of acedapsone instead of dapsone, which was proven effective, but due to administrative difficulties, it could not be taken on a mass scale [2].

Role in the Indian Association of Leprologists (IAL)

Leprosy relief work in India prior to independence was carried out by the British Empire Leprosy Relief Association (BELRA), founded on January 27, 1925; however, after the independence of India, BELRA was renamed as Hind Kusht Nivaran Sangh (Indian Leprosy Association) in 1950. Shortly after, in 1954, the IAL was founded in Madras (now Chennai), and Dr. Dharmendra was elected as its first president and reelected for four further terms [3]. The organization, to this day, works towards the cause of leprosy.

The main objectives of IAL were to (a) promote the study of and research in leprosy, (b) to create public opinion in matters relating to the prevention and cure of leprosy and rehabilitation of patients, and (c) to cooperate with the medical and other regional and national institutions having altogether or in part similar objectives [4].

He was also a diligent editor of the Journal of Leprosy in India, established in 1929 and later renamed by him in 1984 to the Indian Journal of Leprosy. He continued his work well into his 80s with two short breaks in between [5]. To date, the journal continues to be one of the most important publications related to leprosy in the English language.

Role in the National Leprosy Control Programme

On the verge of retirement in 1955, he was asked by the Government of India to head the National Leprosy Control Programme, and he took charge as its first director. Following this, he laid the foundation for one of the most successful health programs in the country [2]. After the inception of the wide use of Multi-Drug Therapy (MDT) in 1983, the national program was revised and renamed the National Leprosy Eradication Programme, which strategized to control the disease through a reduction in the quantum of infection in the general population and reduce the infective sources and ultimately break the chain of disease transmission. It is as a result of this very national program that the Central Leprosy Training and Research Institute (CLTRI) in Chengalpattu, Tamil Nadu, was established [6]. As part of the national program, Leprosy Control Units (LCU) in every district, which catered to one lakh individuals, and Survey Education and Treatment (SET) centers, which catered to 15000 individuals, were established [1].

Work at Central Leprosy Teaching and Research Institute, Chengalpattu

In 1957, under the aegis of the National Leprosy Control Programme, he was tasked with organizing the first institute solely dedicated to leprosy research and education in Chengalpattu. He gathered a group of young medical men and developed them into productive scientists. His study of the effect of dapsone prophylaxis among contacts of lepromatous patients is an excellent illustration of his scientific experiment design, with the population serving as the laboratory. In 1967, he retired as director of the Central Leprosy Teaching and Research Institute but remained an emeritus scientist of the Indian Council of Medical Research (ICMR) until 1970 before moving to New Delhi post-retirement [5].

Books published

**Figure 2:**
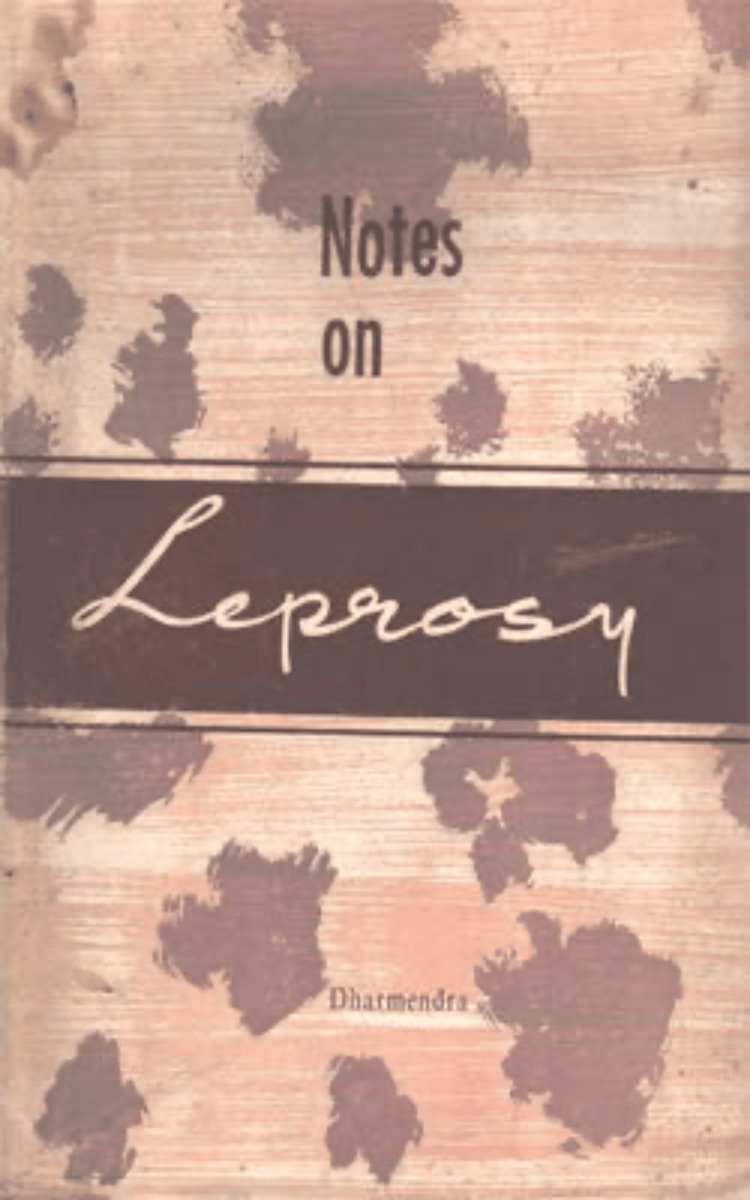
Dr. Dharmendra's book "Notes on Leprosy" published in 1967 Source [7]

Dr. Dharmendra authored the textbook "Notes on Leprosy" (Figure 2) in 1960, and a revised version was released in 1967 [5]. He edited the monumental work "Leprosy," the first volume of which was published in 1978 and the second in 1985, on account of its popularity among leprosy workers [5].

Life after retirement

Dr. Dharmendra had been associated with the Journal of Leprosy in India for a long time. While he was not the head of the department, Dr. Lowe was the journal's editor. When he went on leave, Dr. Dharmendra used to update it under Dr. Lowe's name. After Dr. Lowe moved from the leprosy department to the position of Professor of Tropical Medicine, he became the permanent editor of the journal. He held it until 1957, when he moved to Chengalpattu and left the journal editorship with his successor in Calcutta. However, after two years, the quality of the Journal of Leprosy in India had deteriorated to such an extent that he was asked to resume his position as editor for the remainder of his time there [2].

Advice on future prospects to eradicate leprosy in India

Dr. Dharmendra emphasized four key points for leprosy eradication in India: first, raising the financial position and socioeconomic condition of the general population to end malnutrition; second, curbing unsanitary habits such as blowing the nose and spitting indiscriminately; third, avoiding overcrowding; and fourth, providing short-term isolation of affected individuals to avoid contact with a healthy population [2].

Awards and honors

Dr. Dharmendra was the first Indian to win the prestigious Damien Dutton Award, which honors outstanding service to the cause of leprosy in 1970. He also received Padma Shri [2], the fourth-highest civilian award of the Republic of India, and the International Gandhi Award in 1986 [5].

Demise

After dedicating 60 years of his life to the study and treatment of leprosy, Dr. Dharmendra passed away on March 10, 1991, at the age of 91 [5]. Although he is no longer with us, his pioneering work and unparalleled contributions to leprosy research remain timeless.

## Conclusions

Dr. Dharmendra's legacy extends beyond scientific achievements. He challenged the stigma associated with leprosy and inspired generations of Indian researchers and clinicians to pursue excellence in dermatology and public health. His name is etched in the annals of medical history, and his contributions will be remembered and revered as long as the fight against leprosy continues.
